# Beyond Bell’s: A Case Study of Bilateral Facial Paralysis From Lyme Neuroborreliosis

**DOI:** 10.7759/cureus.100160

**Published:** 2025-12-26

**Authors:** Kainuo Wu, Christopher Peterson, Mariana Gomez de la Espriella, Tasaduq Fazili

**Affiliations:** 1 Internal Medicine, Virginia Tech Carilion School of Medicine, Roanoke, USA; 2 Infectious Diseases, University of North Carolina at Chapel Hill, Chapel Hill, USA; 3 Infectious Diseases, Virginia Tech Carilion School of Medicine, Roanoke, USA

**Keywords:** bilateral facial palsy, borrelia, facial paralysis, lyme disease, neuroborreliosis

## Abstract

Bilateral facial palsy is a rare neurological presentation that often reflects an underlying systemic disease that requires prompt and comprehensive diagnostic evaluation to guide appropriate management.

A 50-year-old man presented with bilateral facial palsy without a recent history of vector exposure or characteristic rash. Extensive diagnostic studies included autoimmune panels, neurovascular and structural imaging, cerebrospinal fluid (CSF) analysis, infectious cultures, and various viral and bacterial serologies. The absence of a clear exposure history and variable latency period following initial exposure contributed to diagnostic uncertainty. Lyme meningitis was confirmed based on CSF findings and a markedly elevated *Borrelia *CSF to serum antibody index after other etiologies were excluded. The patient was treated with seven days of intravenous ceftriaxone and 14 days of oral doxycycline, achieving complete neurological recovery at three months.

This case highlights the exhaustive workup required to identify the cause of bilateral facial palsy. Early specialty consultation is critical to expedite diagnostic workup. Clinicians should maintain a high index of clinical suspicion for Lyme disease despite an absent exposure history or rash. Prompt recognition of bilateral facial palsy and timely intervention are essential to ensure optimal neurological recovery.

## Introduction

Facial nerve palsy, paresis, or paralysis of facial muscle due to dysfunction of the cranial nerve 7 is a common presentation in general medical practice, with an incidence of 20-30 cases per 100,000 per year [1]. It accounts for over 60% of facial paralysis cases, with idiopathic or Bell’s palsy being the most common [1]. Bilateral facial palsy is an extremely rare condition that constitutes 0.3%-2% of all facial palsies with an overall incidence of one per five million per year [1,2]. It is characterized by impairment of bilateral facial muscles innervated by both facial nerves within 30 days of onset on each side. The differential is broad and includes infectious, autoimmune, and traumatic causes. However, there is often a causal etiology as opposed to unilateral facial palsy, where most cases are idiopathic [2,3]. Most etiologies often have a good prognosis; however, delayed diagnosis can lead to devastating consequences. Thus, a comprehensive clinical approach should be adopted to achieve a definitive diagnosis and appropriate treatment.

Lyme neuroborreliosis describes neurological involvement due to systemic infection of the spirochete *Borrelia burgdorferi*, often transmitted by *Ixodes* ticks, which usually manifests weeks to months after the initial onset of target rash and flu-like symptoms. Prognosis is overall favorable, but prompt recognition and treatment are paramount for faster recovery and reducing the risk of residual facial paresis [4]. In this paper, we present a case of bilateral facial nerve palsy as the initial manifestation of Lyme meningitis.

## Case presentation

A 50-year-old Hispanic man presented to the emergency department with three days of posterior headache, dysarthria, bilateral jaw pain, facial droop noted by his partner, and thoracic back pain. On examination, the patient was non-toxic appearing. He was afebrile, borderline tachycardic, with normal respiratory rate, and had a mildly elevated blood pressure of 145/92 mmHg. Neurological examination revealed bilateral facial palsy with a House-Brackmann paralysis scale of 5, evidenced by the inability to raise his eyebrows and smile. He also reported altered taste sensation. The rest of the cranial nerves, sensation, strength, motor, and reflexes were all intact. Brudzinski, Hoffman, Babinski, and Kernig signs were all negative. Admission investigations were notable for leukocytosis of 15,000/µL and hemoglobin of 17 g/dL, with normal platelet count, electrolytes, renal function, and hepatic function. Systemic inflammatory markers, namely, sedimentation rate and C-reactive protein, were undetectable. Computed tomography scan (CT) of the brain, thoracic spine, and maxillofacial region did not show hydrocephalus, subdural effusion, hyperdense lesions, fractures, abscess, discitis, or any other acute abnormalities. Magnetic resonance imaging (MRI) of the brain and magnetic resonance venography (MRV) of the head were negative for meningeal enhancement, cranial lesions, thrombosis, abscess, infarction, or any other abnormalities. Neurology was consulted, and the patient was hospitalized to undergo further testing. Lumbar puncture was performed, and cerebrospinal fluid (CSF) analysis demonstrated a lymphocytic pleocytosis, with white blood cell (WBC) of 415/μL and 85% lymphocytes, red blood cell (RBC) of 0/μL, glucose of 39 mg/dL, and protein of 233 mg/dL (Table 1).

**Table 1:** CSF testing results WBC: white blood cell, RBC: red blood cell, PMN: polymorphonuclear neutrophil, TB: tuberculosis, CSF: cerebrospinal fluid

Category	Test/parameter	Result	Interpretation
Appearance	Color	Colorless	Normal
Cell counts	WBC	451/mm³	↑ Elevated
	RBC	0/mm³	None detected
Differential	PMN	1%	Very low
	Lymphocytes	85%	Predominant
	Mononuclear cells	14%	Minor proportion
Chemistry	Protein	233 mg/dL	↑ High
	Glucose	39 mg/dL	↓ Low
Infectious tests	Cryptococcal antigen (CSF)	Negative	No cryptococcal infection
	Acid-fast stain (CSF)	Negative	No TB organisms seen
	Culture	2+ WBC, no organism	No growth
Lyme disease	Western blot IgG	18 KD, 23 KD, 39 KD, 41 KD, 58 KD	Positive bands
	Western blot IgM	23 KD, 39 KD	Positive bands

Further clinical history revealed that the patient is a truck driver who has been living in the United States for the past 20 years. His last international travel was to El Salvador, his home country, occurring four years ago prior to admission. He denies a history of tick bites, and a comprehensive skin examination did not reveal an identifiable rash. Later in the course, the patient became toxic-appearing and was empirically treated with intravenous acyclovir, ampicillin, ceftriaxone, and linezolid for meningitis. Infectious disease was consulted and recommended a broad infectious workup. Additional CSF studies were obtained, including acid-fast stain, Gram stain, cryptococcal antigen, and fungal culture, which were all negative. Epstein-Barr virus (EBV) serology was positive for IgG and negative for IgM. The serum tick-borne panel, which includes Lyme DNA PCR, was negative. Serum human immunodeficiency virus (HIV) viral load was not detected. Rapid plasma reagin (RPR), herpes simplex virus (HSV) 1 and 2 DNA, and antinuclear antibody (ANA), anti-Ro (SS-A), anti-Jo (SSB), and anti-angiotensin-converting enzyme (ACE) antibodies were all negative. The Lyme Western blot of the CSF eventually returned positive band 23 KD and 39 KD for IgM and band 18KD, 23KD, 39KD, 41KD, and 58KD for IgG, and the Lyme CSF to serum antibody index is 9.53, suggesting intrathecal production of antibodies against *Borrelia*, meeting diagnostic criteria for Lyme meningitis (Table 2). Acyclovir, ampicillin, and linezolid were discontinued, and the patient completed seven days of IV ceftriaxone for neuroborreliosis. He was then switched to oral doxycycline (100 mg, twice daily) at discharge for an additional 14 days to complete 21 total days of treatment. During his hospital stay, he also received 10 doses of 10 mg dexamethasone injections and did not receive additional steroids on discharge. The patient had significant neurological improvement on discharge, and facial palsy resolved three months after leaving the hospital.

**Table 2:** Serological testing results Ab: antibody, Ag: antigen, EBV: Epstein-Barr virus, HIV: human immunodeficiency virus, RPR: rapid plasma reagin, RNA: ribonucleic acid, PCR: polymerase chain reaction, HSV: herpes simplex virus, TB: tuberculosis, ANA: antinuclear antibody, SSA: Sjögren's syndrome-related antigen A, SSB: Sjögren's syndrome-related antigen B, ACE: angiotensin-converting enzyme

Category	Test	Result	Interpretation
Lyme serology	Western blot IgG	18 KD, 23 KD	Positive
	Western blot IgM	23 KD, 39 KD, 41 KD	Positive
CSF:Lyme antibody index	Intrathecal Lyme antibody production	9.53	Strongly positive
EBV serology	Viral capsid Ag IgG	419 U/mL	Past infection
	Viral capsid Ag IgM	<36 U/mL	Negative
	Nuclear Ag IgG	84.5 U/mL	Past exposure
HIV testing	HIV Ag/Ab, fourth generation	Non-reactive	Negative
	HIV RNA PCR	Not detected	No viremia
Syphilis	RPR titer	Non-reactive	No active syphilis
	Treponemal Abs	Reactive minimal → repeat nonreactive	Negative
HSV	HSV1/HSV2 PCR	Not detected	No HSV detected
Hepatitis	Hepatitis C Ab	Non-reactive	Negative
TB	QuantiFERON TB	Negative	No latent/active TB
Autoimmune markers	ANA	Negative	No autoimmune activity
	Sjögren's antibody SSA, SSB	<1.0	Negative
	ACE	22 U/L	Normal
Bacterial antigens	Streptococcus pneumoniae Ag	Negative	No infection
Tick-borne diseases	Tick-borne panel	Not detected	Negative

## Discussion

Facial palsy is a neurological pathology with a wide differential, most often recognized as a cranial nerve VII deficit. Bilateral facial palsy comprises a rare subset of facial palsies, encompassing 0.3%-2% of all facial palsies [5]. Facial palsy is a known complication of Lyme disease due to an inflammatory process (likely autoimmune) of the cranial nerve VII (and not direct invasion of the nerve by *Borrelia*) [6,7]. Bilateral facial palsy is categorized as simultaneous, with both sides developing paralysis within 30 days [8].

It is important to identify the underlying etiology of acute bilateral facial palsy to appropriately triage patients and initiate timely treatment. This is different from unilateral facial palsy, which often does not require laboratory or radiological investigation since the majority of cases are idiopathic [9]. Differential for patients presenting with bilateral facial palsy can be broadly characterized as traumatic, infectious, autoimmune, neoplastic, congenital, neurological, metabolic, iatrogenic, or idiopathic [1,3]. The most common etiologies are autoimmune (especially Guillain-Barré syndrome), infectious, and idiopathic (namely, Bell’s palsy), with Lyme disease being the most common infectious cause of bilateral facial paralysis [4,10]. Life-threatening or highly morbid etiologies such as Guillain-Barré syndrome, head trauma, cerebrovascular accident, leukemia, and meningitis should be ruled out [1,10]. A computed tomography scan is rapid and useful in assessing several immediately threatening etiologies. If CT is unrevealing, MRI should be considered as it allows for better visualization of tumor infiltration, nerve lesions, meningeal inflammation, and changes in the acoustic canal [1]. Diagnostic workup should focus on the most likely etiologies based on history and risk factors. Serum studies should include testing for HIV, syphilis, inflammatory markers (erythrocyte sedimentation and C-reactive protein), Lyme antibodies, and autoimmune markers (such as antinuclear antibodies) [1,11,12]. However, some cases can present without identifiable features in patients’ clinical history. Prior papers highlighted the difficulty of diagnosing autoimmune diseases, with sarcoidosis being a classic example since it is not associated with specific biomarkers and can present with vague clinical signs [3,4]. The authors recommend urgent neurology consult, which was also pursued in this case, as well as a comprehensive diagnostic approach that accounts for the various differentials of bilateral facial palsy. While this case had an identifiable infectious picture, other etiologies were also worked up to rule out high morbid conditions and provide accurate, timely treatment.

This case had a high clinical index of suspicion for meningitis, leading to urgent lumbar puncture and early consultation of neurology. Lyme neuroborreliosis was highly suspected due to the manifestation of bilateral facial palsy and an endemic epidemiological location. However, cerebrospinal fluid analysis should also test for varicella-zoster virus (VZV), HSV, and EBV, which are common sources based on retrospective reviews [13,14]. We note that *Borrelia* PCR has a lower sensitivity, especially in patients presenting less than six weeks from disease onset, and thus, a negative value does not rule out the disease (as seen here) [15]. The CSF result was consistent with lymphocytic pleocytosis with WBC of 415/μL and 85% lymphocytes, glucose of 39 mg/dL, and protein of 233 mg/dL. The markedly elevated CSF to serum antibody for *Borrelia* confirmed intrathecal production of antibodies. A diagnosis of Lyme neuroborreliosis was confirmed based on the above findings and after alternative etiologies returned negative [15]. While tick exposure should be determined in patients in Lyme endemic areas, only a minority of patients with Lyme disease recall tick exposure [16]. Expert consultation from neurology and, depending on suspected etiology, infectious diseases, rheumatology, and otolaryngology should be obtained to expedite workup for acute bilateral facial palsy [3,4], due to the wide differential and potential for life-threatening pathologies with bilateral facial palsy (unlike unilateral facial palsy) [1]. As seen in this case and prior studies [9,17], subjective reports of tick exposure are not reliable, and transient target lesions (erythema migrans) can easily be missed. The manifestation of facial palsy can occur at various time courses of Lyme disease, and patients and clinicians may not readily correlate tick exposure to neurological symptoms [17]. The most recent guideline for the prevention, diagnosis, and treatment of Lyme disease integrates expert opinions from the Infectious Diseases Society of America (IDSA), American Academy of Neurology (ANN), and American College of Rheumatology (ACR), underscoring the importance of a multidisciplinary approach [18]. This case emphasizes the importance of early subspecialty consultation to investigate etiologies that may not be routinely encountered by the general internist [17,19].

Treatment for Lyme disease with facial palsy typically involves oral doxycycline; the IDSA recommends IV ceftriaxone, penicillin G, cefotaxime, or oral doxycycline for 14-21 days in the setting of central or peripheral nervous system complications from Lyme disease [18]. While steroids are typically given for Bell’s palsy, their usefulness for facial palsy due to Lyme disease is unclear [20]. However, the IDSA makes no recommendation for or against using steroids in addition to antibiotics, citing limited evidence [18]. Studies have failed to show a clear benefit, although steroids do not appear to worsen illness either [17]. Most patients with Lyme disease with bilateral facial palsy have complete resolution of cranial nerve VII palsy, often within one month [10]. Some studies note that those with bilateral facial palsy have a higher risk of neurological sequelae, while others observed a similar rate of recovery [2,10].

## Conclusions

Although bilateral facial palsy is diagnosed clinically like unilateral facial palsy, bilateral facial palsy is a sign of an underlying systemic condition that requires exhaustive workup and urgent medical intervention. The presence of posterior headache, dysarthria, and rapid-onset facial palsies in the endemic region on initial presentation raised concern for Lyme meningitis despite absent exposure history or rash. Aside from serological testing for various infectious causes and autoimmune biomarkers, urgent lumbar puncture and neuroimaging must be considered to achieve a definitive diagnosis. Early involvement of consultants, including neurology and infectious disease, is critical to expedite workup. The positive Lyme Western blot in the CSF and significantly elevated CSF to serum antibody index confirmed the diagnosis of Lyme neuroborreliosis. Bilateral facial palsy is often associated with higher rates of long-term sequelae than unilateral facial palsy. Early recognition and treatment with intravenous ceftriaxone followed by extended oral doxycycline are paramount to optimize neurological outcome, which would significantly impact patients’ quality of life. This case highlights the acuity of bilateral facial palsy, early subspecialty consultation to guide thorough workup, and impact on long-term neurological sequelae with timely treatment.
